# Procedural success of transcatheter annuloplasty in ventricular and atrial functional tricuspid regurgitation

**DOI:** 10.3389/fcvm.2023.1189920

**Published:** 2023-08-07

**Authors:** Fabian Barbieri, Isabel Mattig, Niklas Beyhoff, Tharusan Thevathasan, Elena Romero Dorta, Carsten Skurk, Karl Stangl, Ulf Landmesser, Mario Kasner, Henryk Dreger, Markus Reinthaler

**Affiliations:** ^1^ Deutsches Herzzentrum der Charité, Department of Cardiology, Angiology and Intensive Care Medicine, Berlin, Germany; ^2^ DZHK (German Centre for Cardiovascular Research), Partner Site Berlin, Berlin, Germany; ^3^ Berlin Institute of Health at Charité—Universitätsmedizin Berlin, BIH Biomedical Innovation Academy, Berlin, Germany; ^4^ Institute of Medical Informatics, Charité—Universitätsmedizin Berlin, Berlin, Germany; ^5^ Institute of Active Polymers and Berlin-Brandenburg Center for Regenerative Therapies, Helmholtz-Zentrum Hereon, Teltow, Germany

**Keywords:** atrial functional tricuspid regurgitation, ventricular functional tricuspid regurgitation, transcatheter annuloplasty, Cardioband®, interventional echocardiography

## Abstract

**Background:**

Transcatheter annuloplasty is meant to target annular dilatation and is therefore mainly applied in functional tricuspid regurgitation (TR). Due to recent recognition of varying disease pathophysiology and differentiation of ventricular and atrial functional TR (VFTR and AFTR), comparative data regarding procedural success for both disease entities are required.

**Methods:**

In this consecutively enrolled observational cohort study, 65 patients undergoing transcatheter annuloplasty with a Cardioband® device were divided into VFTR (*n* = 35, 53.8%) and AFTR (*n* = 30, 46.2%). Procedural success was assessed by comparing changes in annulus dilatation, vena contracta (VC) width, effective regurgitation orifice area (EROA), as well as reduction in TR severity.

**Results:**

Overall, improvement of TR by at least two grades was achieved in 59 patients (90.8%), and improvement of TR by at least three grades was realised in 32 patients (49.2%). Residual TR of ≤2 was observed in 52 patients (80.0%). No significant differences in annulus diameter reduction [VFTR: 11 mm (9–13) vs. AFTR: 12 mm (9–16), *p* = 0.210], VC reduction [12 mm (8–14) vs. 12 mm (7–14), *p* = 0.868], and EROA reduction [0.62 cm^2^ (0.45–1.10) vs. 0.54 cm^2^ (0.40–0.70), *p* = 0.204] were reported. Improvement by at least two grades [27 (90.0%) vs. 32 (91.4%), *p* = 1.0] and three grades [14 (46.7%) vs. 18 (51.4%), *p* = 0.805] was similar in VFTR and AFTR, respectively. No significant difference in the accomplishment of TR grade of ≤2 [21 (70.0%) vs. 31 (88.6%), *p* = 0.118] was noted.

**Conclusion:**

According to our results from a real-world scenario, transcatheter annuloplasty with the Cardioband® device may be applied in both VFTR and AFTR with evidence of significant procedural TR reduction.

## Introduction

Tricuspid regurgitation (TR) is on the verge to become one of the major valvular heart diseases in high-income countries equally affecting morbidity and mortality (1, 2). Epidemiological data have shown a prevalence of significant TR in up to 2.7% of adult patients and were able to reveal an association with increasing age, irrespective of gender (1). Recent studies, which proposed new grading schemes, also revealed the prognostic implications by describing increased mortality rates in torrential TR compared with the ones already immanent in severe forms (3, 4). On the other hand, even mild TR was also associated with an elevated risk for mortality (5).

Although unappreciated for a long time due to poor surgical outcomes with high periprocedural mortality, recent advancements in interventional therapy have shed new light on different pathophysiologies driving disease progression as they also seem to further impact mortality (6, 7). Major factors contributing to the increasing number of patients suffering from functional TR are atrial fibrillation and left-sided heart failure. Nearly one-third of patients with new-onset atrial fibrillation were found to develop significant TR in a population-based study, also affecting their outcome (8). Similar observations were made in large-scale cohorts for heart failure, although the prevalence was lower during follow-up (9). These observations have led to novel sub-classifications, which stand in analogy to the ones for secondary mitral regurgitation, with adherent prognostic relevance (10–12). Yet, only limited data on the different subtypes of functional TR and their effect on interventional valve therapy exist. At present, a single publication investigating the impact of atrial functional TR (AFTR) on procedural outcome with transcatheter edge-to-edge repair (TEER) is found (11). Another option for therapy of TR is transcatheter annuloplasty by using the Cardioband® (Edwards Lifesciences, Irvine, CA, USA) system. The concept to treat annular dilatation, the dominant factor for development of AFTR, would presume that this patient cohort may respond better to therapy by improved procedural outcomes compared with patients suffering from ventricular functional TR (VFTR) (12, 13). As this concept is still unproven, we set out to investigate procedural results of patients undergoing transcatheter annuloplasty for secondary TR according to their aetiology in a consecutively enrolled observational study cohort.

## Material and methods

### Study design and population

This observational multicentre study was conducted at two tertiary care facilities of the Charité—Universitätsmedizin Berlin (Campus Benjamin Franklin and Campus Mitte) in Germany. All patients, who received catheter-based annuloplasty for symptomatic TR, have been screened and consecutively enrolled since the beginning of the dedicated institutional TR program in 2019. Overall, 67 patients were treated based on interdisciplinary heart team decision between February 2019 and January 2023. One patient was excluded from analysis due to a predominantly primary TR with an anterior leaflet prolapse, while another patient was excluded due to a mixed aetiology of TR. Patients with simultaneous finding of severe mitral regurgitation and TR received treatment for mitral regurgitation first and then transcatheter annuloplasty for TR at a later stage (>2 months later). The Institutional Review Board/ethics committee approved the conduction of this trial at each campus (EA4/013/21 and EA1/005/23).

### Echocardiography

Transthoracic and transoesophageal echocardiographies were conducted in every patient as part of routine clinical examination to evaluate the necessity and feasibility of tricuspid valve intervention. Left ventricular ejection fraction (LVEF), tricuspid annular plane systolic excursion (TAPSE), fractional area change (FAC), tenting height, and the calculated systolic pulmonary artery pressure were all assessed during transthoracic echocardiography (14). Tenting height of the tricuspid valve was measured in the apical four-chamber view (11).

Annulus measurements and assessment of TR severity [including vena contracta (VC) width and effective regurgitation orifice area (EROA)] were performed at the beginning and end of each procedure by transoesophageal echocardiography in the presence of general anaesthesia. Grading of TR severity was conducted according to recently proposed algorithms ranging from “none/trace” to “torrential” (3). To respect the individuality of both disease entities, AFTR and VFTR, measurements of the tricuspid annulus were taken in a biplane view [anterior to posterior (mid-oesophageal short-axis view), septal to lateral (RV inflow view)]. Presented values reflect the mean of both measurements. The VC width and the proximal isovelocity surface area (PISA) radius were assessed in a similar manner (15).

### Definition of aetiology

**Figure 1:**
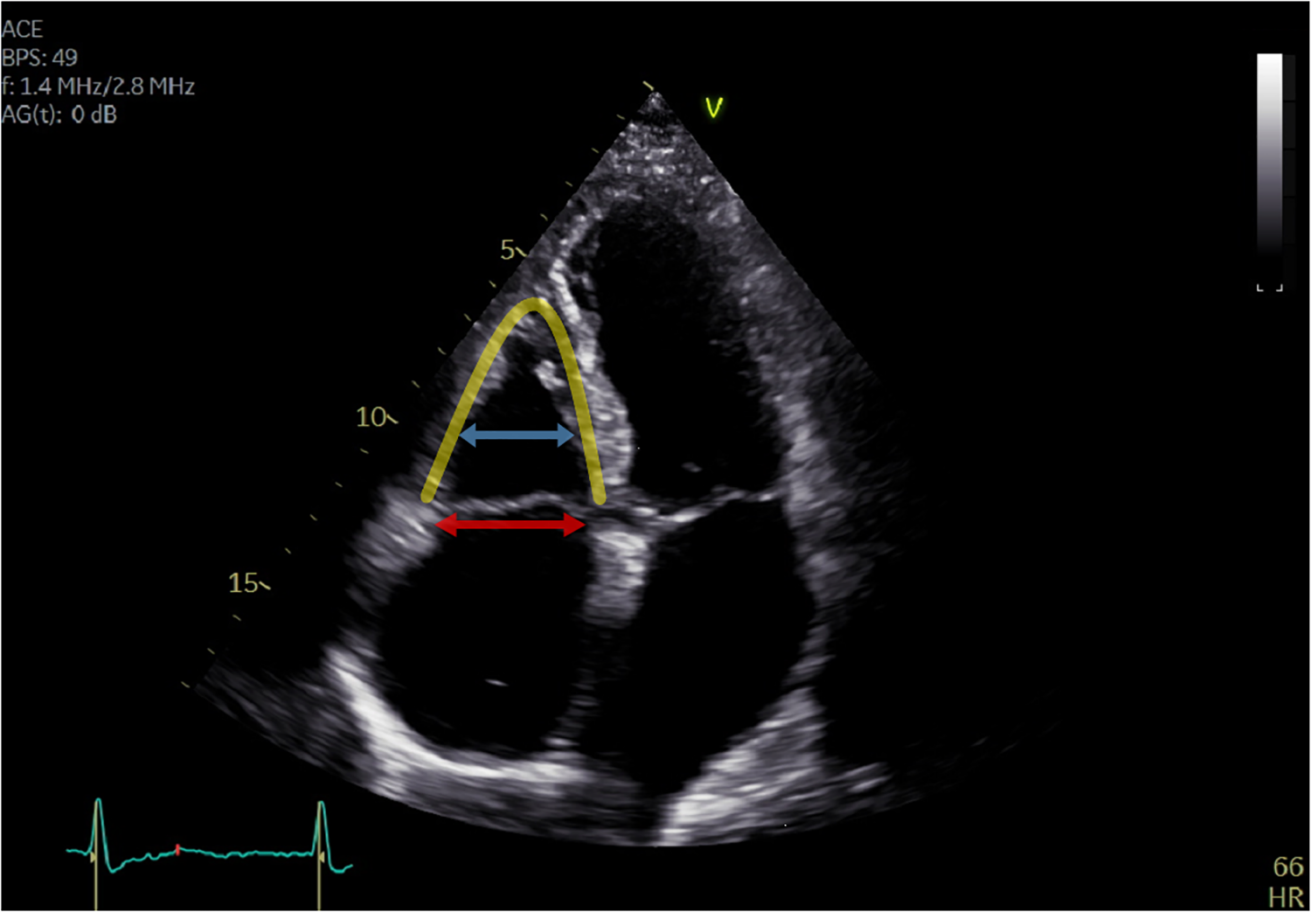
Apical four-chamber view of a patient with atrial functional tricuspid regurgitation. Tricuspid regurgitation is mainly caused by right atrial enlargement with consequent tricuspid annulus dilatation (red arrow). The right ventricle is triangular shaped (yellow line) without remodelling or dilatation (blue arrow), and the coaptation line is found at the height of the tricuspid annulus (<10 mm tenting height).

**Figure 2:**
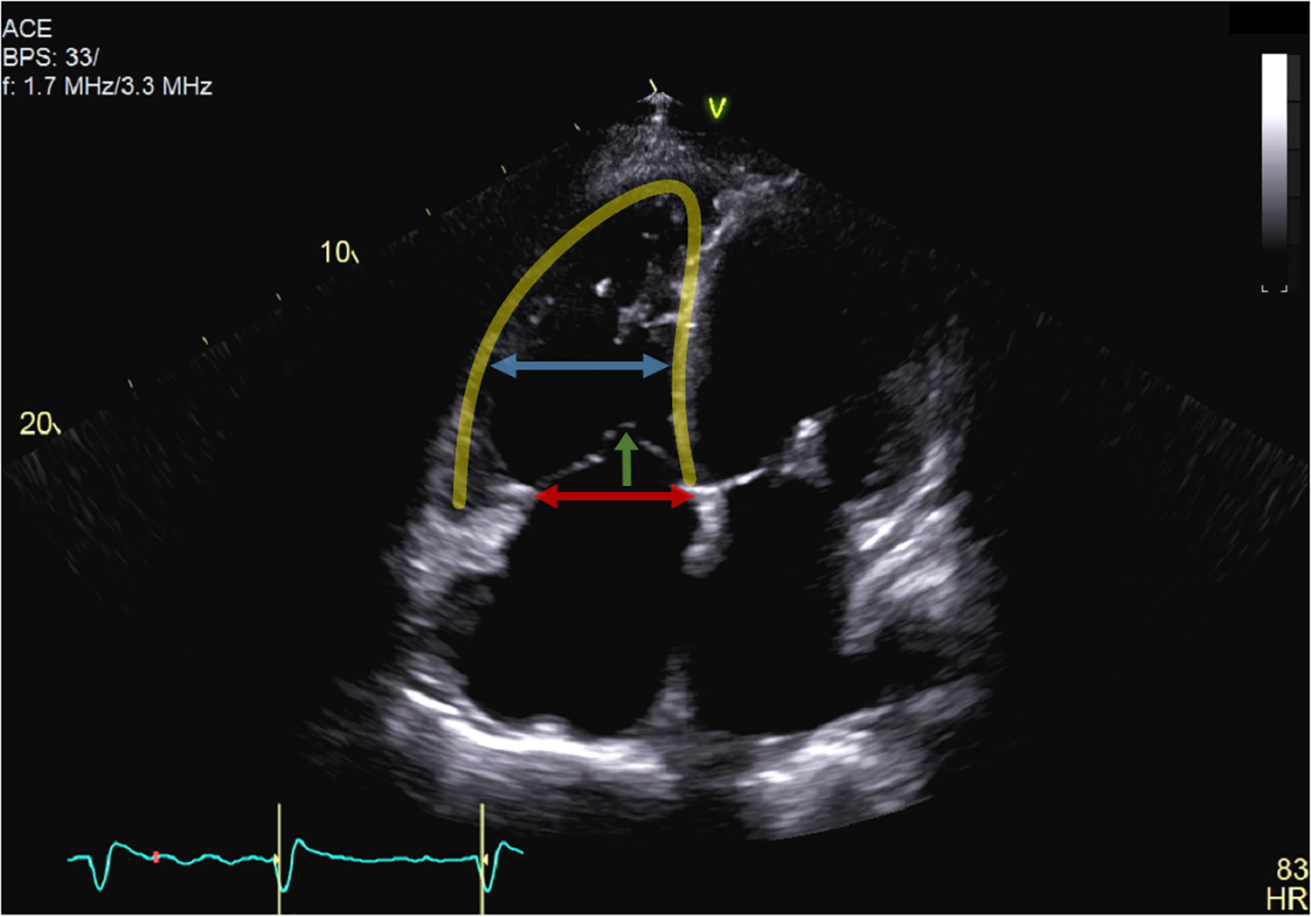
Apical four-chamber view of a patient with ventricular functional tricuspid regurgitation. Tricuspid regurgitation is mainly driven by severe leaflet tethering (green arrow) due to right ventricular dilatation (yellow line) and remodelling. The mid-right ventricular dilatation (blue arrow) was therefore more pronounced than tricuspid annulus dilatation (red arrow), and the line of coaptation was below the tricuspid annulus (>10 mm tenting height).

Aetiology of TR was classified either as primary, cardiac implantable electronic device (CIED)-related, atrial functional, ventricular functional, or mixed. Primary TR was defined by the presence of organic abnormalities to valve morphology, such as prolapse or flail leaflet, resulting in malcoaptation or leaflet deficiency. TR was declared as CIED-related, whenever leaflet impingement or perforation by pacemaker or defibrillator leads was observed. Differentiation of functional TR was achieved by an integrative assessment of multiple parameters. TR was classified as AFTR, whenever right atrial enlargement with consequent tricuspid annulus dilatation was suspected as the main driver for development of TR. These patients typically yielded preserved right ventricular (RV) function, absence of severe RV remodelling (triangular-shaped), and no or only mildly elevated pulmonary artery pressures (Figure 1). On the other hand, VFTR was defined by the presence of RV dysfunction with RV remodelling (midventricular RV dilatation more pronounced than tricuspid annulus dilatation) and corresponding leaflet tethering or tenting (>10 mm). A line of coaptation was therefore typically found below the tricuspid annulus (Figure 2). TR was assumed to be of mixed aetiology, if two or more patterns were found to be of equal dominance (11–13).

### Statistical analyses

Continuous variables were described as median and interquartile range and categorical variables as number and percentage. Distribution of continuous variables was assessed by the Shapiro–Wilk test and inspection of quantile–quantile plots. Calculation of differences in continuous variables was either conducted by *t*-tests or Mann–Whitney *U* tests, according to their distribution. Differences between categorical variables were assessed by the Chi square or Fisher's exact test, whenever applicable. Univariate calculations for good response to therapy (improvement by at least three grades) were conducted by using the same tests. Figures were designed with GraphPad PRISM version 5 (GraphPad Software, Inc., La Jolla, CA, USA), and statistical analyses were conducted by using IBM SPSS version 21 (IBM Corporation, Armonk, NY, USA). *p*-Values of 0.05 or less were considered significant.

## Results

### Baseline characteristics

All baseline characteristics are displayed in Table 1. Out of the 65 patients analysed, 35 (53.8%) were classified as VFTR, whereas 30 (46.2%) fulfilled the criteria for AFTR. Almost two-thirds of the cohort (*n* = 46, 63.1%) were female with a median age of 80 (interquartile range: 77–83) years. All enrolled patients were found to be symptomatic with dyspnoea at exertion being the most relevant clinical symptom (New York Heart Association class II: *n* = 12, 18.5%; class III: *n* = 49, 75.4%; class IV: *n* = 4, 6.2%). The median LVEF was 53% (46–61), while TAPSE and FAC, as markers for right heart function, were 19 mm (17–22) and 37.5% (33.7–41.7), respectively. Overall, the tenting height was 6 mm (3–11), and the calculated systolic pulmonary pressure was 45 mmHg (38–56).

**Table 1:** Baseline characteristics.

Variable	Overall (*n *= 65)	Ventricular functional TR (*n *= 35)	Atrial functional TR (*n *= 30)	*p*-Value
Age (years)	80 (77–83)	80 (77–84)	80 (76–83)	0.989
Gender (female), *n* (%)	41 (63.1)	19 (63.3)	22 (62.9)	1.000
History of peripheral oedema, *n* (%)	46 (70.8)	23 (76.7)	23 (65.7)	0.416
History of ascites, *n* (%)	9 (13.8)	4 (13.3)	5 (14.3)	1.000
New York Heart Association class, *n* (%)				0.064
II	12 (18.5)	4 (13.3)	8 (22.9)	
III	49 (75.4)	22 (73.3)	27 (77.1)	
IV	4 (6.2)	4 (13.3)	0	
EuroSCORE2	4.25 (3.08–7.55)	5.07 (3.45–11.14)	3.99 (2.90–5.77)	0.074
Left ventricular ejection fraction (%)	53 (46–61)	50 (43–59)	56 (50–61)	0.095
Tricuspid annular plane systolic excursion (mm)	19 (17–22)	18 (14–21)	20 (18–22)	**0**.**017**
Right atrial volume (ml)	142 (108–176)	148 (115–181)	142 (96–173)	0.594
Diastolic right ventricular area (cm^2^)	22.8 (18.7–29.8)	24.9 (20.3–34.7)	21.9 (17.3–26.3)	**0**.**037**
Systolic right ventricular area (cm^2^)	14.7 (11.4–17.3)	16.2 (13.7–22.8)	12.9 (10.7–16.1)	**0**.**001**
Fractional area change (%)	37.5 (33.7–41.7)	33.7 (28.0–38.3)	39.3 (36.7–43.6)	**<0**.**001**
Midventricular right ventricular diameter (mm)	35.5 (29.8–43.3)	37.5 (33.0–47.8)	33.0 (27.8–40.5)	**0**.**012**
Calculated systolic pulmonary artery pressure (mmHg)	45 (38–56)	51 (43–60)	43 (37–52)	**0**.**049**
Tricuspid valve tenting (mm)	6 (3–11)	11 (6–13)	3 (2–5)	**<0**.**001**
Cardiac implantable electronic device leads, *n* (%)	10 (15.4)	5 (17.2)	5 (14.3)	1.000
Previous interventional therapy of mitral valve, *n* (%)	14 (21.5)	8 (26.7)	6 (17.1)	0.352
N-terminal pro-natriuretic peptide (ng/L)	2,511 (1,757–4,263)	2,981 (1,556–5,332)	2,500 (1,876–3,603)	0.462
Gamma-glutamyl transferase (U/L)	91 (51–167)	86 (51–175)	91 (47–156)	0.599
Alanine aminotransferase (U/L)	19 (16–24)	19 (13–23)	20 (17–26)	0.325
Aspartate aminotransferase (U/L)	32 (26–38)	30 (26–36)	35 (27–42)	0.185
Creatinine (mg/dl)	1.29 (1.01–1.55)	1.32 (1.20–1.80)	1.25 (0.93–1.46)	0.115
Estimated glomerular filtration rate (ml/min/1.73 m^2^)	45 (35–58)	40 (26–52)	49 (37–60)	0.058
Medication				
Betablocker, *n* (%)	58 (89.2)	25 (83.3)	33 (94.3)	0.234
ACE inhibitor/ARB/ARNI, *n* (%)	49 (75.4)	24 (80.0)	25 (71.4)	0.566
Sodium glucose-linked transporter 2, *n* (%)	16 (24.6)	6 (20.0)	10 (28.6)	0.566
Loop diuretic, *n* (%)	61 (93.8)	28 (93.3)	33 (94.3)	1.000
Thiazide, *n* (%)	17 (26.2)	9 (30.0)	8 (22.9)	0.579
Aldosterone antagonist, *n* (%)	37 (56.9)	17 (56.7)	20 (57.1)	1.000

Numbers are presented as median (interquartile range) or number of patients (percentage).

ACE, angiotensin-converting enzyme; ARB, angiotensin receptor blocker; TR, tricuspid regurgitation.

Values in bold have reached statistical significance (*p* ≤ 0.05).

Differences in baseline criteria between VFTR and AFTR were mainly restricted to echocardiographic parameters. RV function was significantly better in AFTR [TAPSE: 18 mm (14–21) vs. 20 mm (18–22), *p* = 0.017; FAC: 33.7% (28.0–38.3) vs. 39.3% (36.7–43.6), *p* < 0.001], respectively. Furthermore, RV area measurements were significantly enlarged in patients with VFTR [diastolic RV area: 24.9 cm^2^ (20.3–34.7) vs. 21.9 cm^2^ (17.3–26.3), *p* = 0.037; systolic RV area: 16.2 cm^2^ (13.7–22.8) vs. 12.9 cm^2^ (10.7–16.1), *p* = 0.001]. The midventricular RV diameter was more pronounced in VFTR [37.5 mm (33.0–47.8) vs. 33.0 mm (27.8–40.5) *p* = 0.012). No difference in right atrial volume between both groups (*p* = 0.594) was seen. As expected, the calculated systolic pulmonary artery pressure was significantly higher in VFTR [51 mmHg (43–60) vs. 43 mmHg (37–52), *p* = 0.049], while the tenting height was significantly lower in AFTR [11 mm (6–13) vs. 3 mm (2–5), *p* < 0.001]. No significant differences regarding the symptomatic state of the patients as well as medical pre-treatment were observed.

### Procedure-associated data

**Figure 3:**
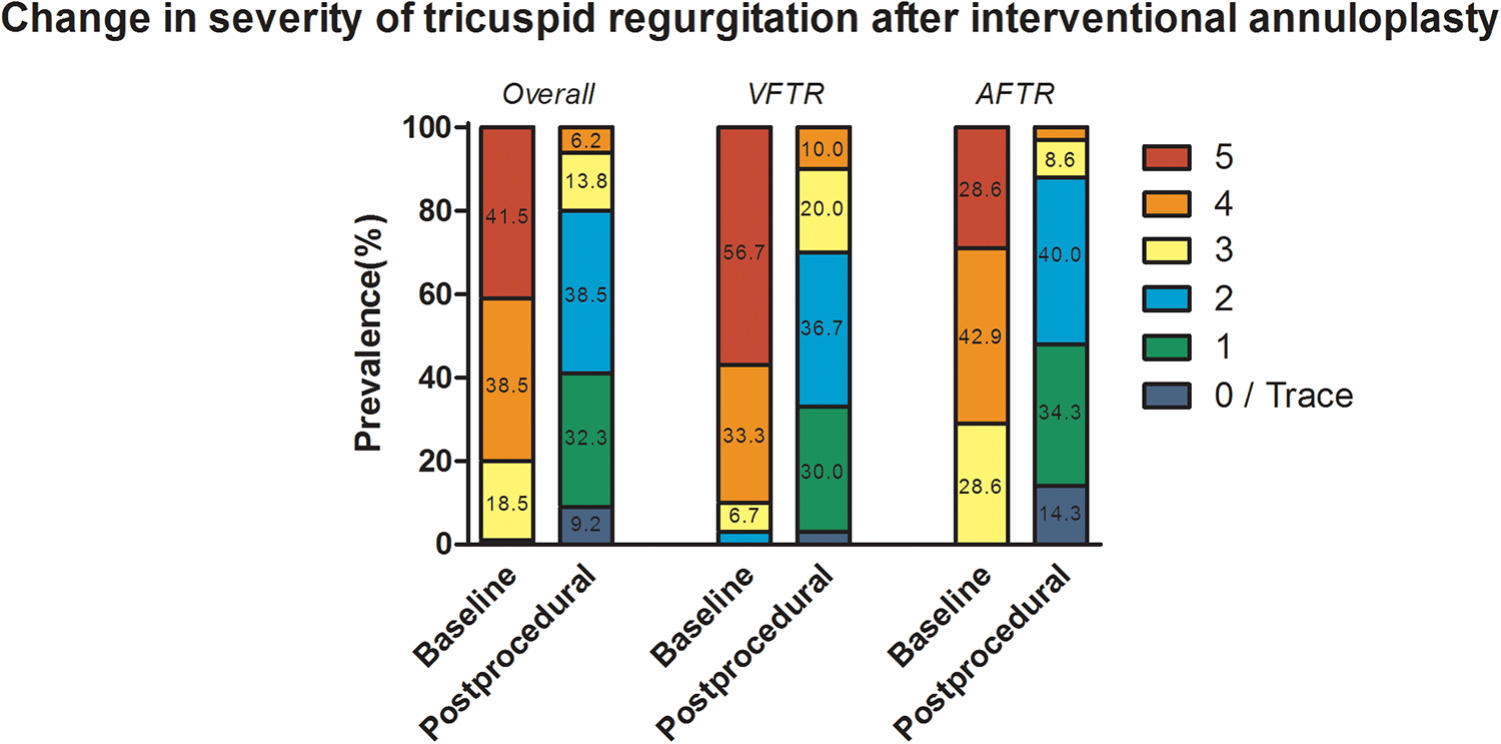
Procedural success displayed as change in severity of tricuspid regurgitation after transcatheter annuloplasty. Numbers in each column present the percentage of prevalence, and values smaller than 5% are not displayed due to limitation of space.

Procedure-associated data and their differences between VFTR and AFTR are displayed in Table 2. Overall, the transoesophageal echocardiography demonstrated significant annulus dilatation [43 mm (40–46)] in all patients. The majority of the patients were either found to have massive (*n* = 25, 38.5%) or torrential TR (*n* = 27, 41.5%) with accordance to the VC width [15 mm (12–20)] and EROA [0.74 cm^2^ (0.60–1.30)]. Cut-to-suture time was 169 min (144–230), and fluoroscopy time was 3,770 s (2,672–4,456). Implantation of the device was successful in all but one patient (*n* = 64, 98.5%), in whom implantation was not attempted after right coronary artery dissection at the beginning of the procedure requiring cardiopulmonary resuscitation. Persistent injury to the right coronary artery (either stenosis or perforation) necessitating percutaneous intervention occurred in three (4.6%) patients and transient bradycardia in four (6.2%) patients. One (1.5%) major access site complication led to postponement of Cardioband® implantation. Improvement by at least two grades was achieved in 59 (90.8%) patients, and improvement by at least three grades was realised in 32 (49.2%) patients. TR of grade 2 or less was accomplished in 52 (80%) of patients (Figure 3). No case of periprocedural mortality was found.

**Table 2:** Intraprocedural data.

Variable	Overall (*n *= 65)	Ventricular functional TR (*n *= 35)	Atrial functional TR (*n *= 30)	*p*-Value
Annulus diameter baseline (mm)	43 (40–46)	42 (39–44)	43 (40–49)	**0**.**050**
Annulus diameter postprocedural (mm)	30 (27–35)	31 (29–33)	29 (27–36)	0.818
Δ annulus diameter (mm)	12 (9–13)	11 (9–13)	12 (9–16)	0.210
Vena contracta width baseline (mm)	15 (12–20)	17 (15–20)	13 (12–19)	0.067
Vena contracta postprocedural (mm)	5 (3–6)	5 (4–7)	3 (2–6)	**0**.**034**
Δ vena contracta (mm)	12 (7–14)	12 (8–14)	12 (7–14)	0.868
Effective regurgitation orifice area baseline (cm^2^)	0.74 (0.60–1.30)	0.93 (0.70–1.50)	0.64 (0.54–0.90)	**0**.**011**
Effective regurgitation orifice area postprocedural (cm^2^)	0.20 (0.10–0.30)	0.29 (0.18–0.48)	0.19 (0.04–0.29)	**0**.**029**
Δ effective regurgitation orifice area (cm^2^)	0.58 (0.44–0.77)	0.62 (0.45–1.10)	0.54 (0.40–0.70)	0.204
Improvement by at least two grades, *n* (%)	59 (90.8)	27 (90.0)	32 (91.4)	1.000
Improvement by at least three grades, *n* (%)	32 (49.2)	14 (46.7)	18 (51.4)	1.000
TR of ≤2 postprocedural, *n* (%)	52 (80.0)	21 (70.0)	31 (88.6)	0.118
Device length				**0**.**029**
C	2 (3.1)	1 (3.5)	1 (2.9)	
D	7 (10.9)	5 (17.2)	2 (5.7)	
E	20 (31.3)	13 (44.8)	7 (20.0)	
F	35 (54.7)	10 (34.5)	25 (71.4)	
Cut-to-suture time (minutes)	169 (144–230)	194 (155–236)	158 (136–220)	0.200
Fluoroscopy time (seconds)	3,770 (2,672–4,456)	3,881 (3,134–4,738)	3,410 (2,544–4,433)	0.095
Radiation exposure (grey/cm^2^)	67.9 (45.7–123.9)	74.8 (52.6–158.1)	67.9 (38.8–106.3)	0.257

Numbers are presented as median (interquartile range) or number of patients (percentage).

Δ, delta; TR, tricuspid regurgitation.

Values in bold have reached statistical significance (*p* ≤ 0.05).

Patients classified as AFTR were found to have numerically higher dilated annuli [42 mm (39–44) vs. 43 mm (40–49), *p* = 0.050] and significantly reduced EROA [0.93 cm^2^ (0.70–1.50) vs. 0.64 cm^2^ (0.54–0.90), *p* = 0.011]. Nonetheless, there were no significant differences in annulus diameter reduction [11 mm (9–13) vs. 12 mm (9–16), *p* = 0.210], VC reduction [12 mm (8–14) vs. 12 mm (7–14), *p* = 0.868], and EROA reduction [0.62 cm^2^ (0.45–1.10) vs. 0.54 cm^2^ (0.40–0.70), *p* = 0.204]. Furthermore, no differences regarding cut-to-suture time (*p* = 0.200), fluoroscopy time (*p* = 0.095), and radiation exposure (*p* = 0.257) were reported. Improvement by at least two grades [27 (90.0%) vs. 32 (91.4%), *p* = 1.000] and three grades [14 (46.7%) vs. 18 (51.4%), *p* = 0.805] were similar in VFTR and AFTR, respectively. No significant difference in the accomplishment of TR grade of 2 or less [21 (70.0%) vs. 31 (88.6%), *p* = 0.118; Figure 3] was noted.

### Predictors of good response to therapy

To assess predictors for good response to therapy, multiple parameters were investigated regarding their predictive value for TR reduction by at least three grades. Unfortunately, neither female sex (*p* = 0.612), LVEF (*p* = 0.675), TAPSE (*p* = 0.440), right atrial volume (*p* = 0.277), diastolic RV area (*p* = 0.516), systolic RV area (*p* = 0.624), FAC (*p* = 0.593), calculated systolic pulmonary artery pressure (*p* = 0.572), tenting height (*p* = 0.756), CIED leads (*p* = 0.302), nor annulus diameter (*p* = 0.702) were found to predict a good procedural outcome.

## Discussion

Over the past few years, several catheter-based technologies targeting TR have become available (16). Yet, the question, which device to use in an individual patient to ensure an optimal result, remains unsolved. In functional TR, reversal of annular dilatation may constitute a key component not only in surgical but also in transcatheter-based repair. As both, atrial and ventricular enlargement may cause annular dilatation, this study ought to investigate transcatheter annuloplasty with the Cardioband® device for both aetiologies.

Currently, there is only a single publication, which has investigated procedural results of tricuspid valve intervention distinguished by aetiology (11). Similar to our results, Schlotter et al. did not observe any difference in procedural success (AFTR: 86% with TR of ≤2, non-AFTR: 81% with TR of ≤2) while applying TEER. Therefore, the detected difference in mortality was rather attributed to the underlying disease, than improvement in TR, also leading to a new definition of AFTR.

Our findings regarding the procedural success in treatment with transcatheter annuloplasty are in line with this study. Even though patients suffering from VFTR were found to have more pronounced forms of TR at baseline, as also expressed by baseline EROA, no significant difference was observed in the changes of VC width and EROA due to transcatheter annuloplasty. The initial difference in severity might also explain the tendency of more frequently achieving moderate or less TR in AFTR. Yet, no discrepancy in reduction by at least two or three grades between both aetiologies was determined. Baseline characteristics significantly differed in terms of RV function, volumes, pulmonary artery pressure, and leaflet tenting height in our cohort, which are by definition ought to be more impaired in VFTR. Furthermore, none of these parameters independently predicted the procedural results, supporting equal efficiency of transcatheter annuloplasty in both cohorts.

In contrast to ventricular dilatation causing annular enlargement and leaflet tethering in the VFTR cohort, the term AFTR suggests a dilatation of the right atrium as a major component in the development of TR. However, right atrial volumes were comparable in both groups, supporting the idea of an existing continuum between both entities. Since impairment of RV function alone will almost certainly increase atrial volumes, an atrial component of TR will be present in most of VFTR patients.

From a pathophysiological point of view, and as demonstrated by our data, direct annuloplasty is able to reverse annular dilation, but not leaflet tethering as the second component of TR. According to these considerations, we expected more pronounced TR reduction with the Cardioband® device in the AFTR cohort. In fact, in terms of postprocedural VC, AFTR actually showed better results compared with their ventricular functional counterparts. However, as already mentioned earlier, this result has to be interpreted in the context of a trend towards increased VC values at baseline in the VFTR group. As stated above, the total VC reduction after the procedure was comparable in both groups. It is also important to mention that mid- and long-term postprocedural remodelling may differently behave in VFTR and AFTR. Therefore, investigation of follow-up data may be crucial to better clarify the effect of annuloplasty in both cohorts.

Overall, a reduction in TR severity of at least two degrees was achieved in more than 90% of the cohort. Although 80% of the cohort presented with advanced stages of the disease displaying massive or torrential TR, a TR reduction to moderate TR or less after the procedure was still accomplished in 80% of the patients. These results are comparable to recent investigations, which first described the procedural success of interventional annuloplasty (17). Despite the fact that only procedural success is reported, mid-term persistence of obtained results was already shown by multiple observational trials (18, 19). The causality of symptomatic improvement due to TR reduction was recently described by the TRILUMINATE Pivotal trial (20). Nonetheless, further studies will be needed to investigate the prognostic impact of TR reduction. It remains to be shown, which clinical and echocardiographic parameters are useful to better identify the patients benefiting from interventional valve therapy. In that regard, the concept of proportional and disproportional functional TR seems to be promising (21).

## Limitations

Despite lack of follow-up data, this study represents a hypothesis-generating investigation in the largest so far reported patient cohort addressing the procedural outcome after Cardioband® in AFTR and VFTR patients. It remains a major difficulty to differentiate later stages of atrial functional TR, which also include RV remodelling due to permanent volume overload of the right heart. Regarding our study, these patients were classified as VFTR. Furthermore, the presence of significant TR may lead to an underestimation of calculated pulmonary artery pressure. Other limitations include the relative small sample size, the absence of mid-term outcome as well as any functional or mortality data, and a lack of a standardised algorithm to classify both forms of functional TR.

## Conclusion

In conclusion, this is the first analysis showing that direct tricuspid annuloplasty might be able to reverse annular dilatation in AFTR and VFTR to a similar extent with a significant decrease in TR severity. We therefore conclude that transcatheter annuloplasty with the Cardioband® device may be applied in both groups of patients with significant evidence of procedural TR reduction according to our results from a real-world scenario. However, further studies including follow-up investigations are necessary to confirm our results.

## Data Availability

The raw data supporting the conclusions of this article will be made available by the authors upon request, without undue reservation.
